# Comparison of External Ureteral Catheter and Double-J stent as Drainage Methods for Tubeless Percutaneous Nephrolithotomy: A Systematic Review and Meta-Analysis

**DOI:** 10.1590/S1677-5538.IBJU.2024.0356

**Published:** 2025-01-10

**Authors:** Clarissa Tania, Edwin Tobing, Christiano Tansol

**Affiliations:** 1 Universitas Padjadjaran Hasan Sadikin Hospital Department of Urology West Java Indonesia Department of Urology, Hasan Sadikin Hospital, Universitas Padjadjaran, West Java, Indonesia; 2 Universitas Pelita Harapan Faculty of Medicine Department of Urology Banten Indonesia Department of Urology, Faculty of Medicine, Universitas Pelita Harapan, Tangerang, Banten, Indonesia

**Keywords:** Kidney Calculi, Nephrolithotomy, Percutaneous, Urinary Catheters

## Abstract

**Purpose:**

The external ureteral catheter (EUC) and double-J stent (DJ-stent) are frequently used for drainage in tubeless percutaneous nephrolithotomy (PCNL). This study aims to compare the outcomes and effectiveness of these two methods.

**Materials and Methods:**

We conducted a detailed literature search using relevant key words on Google Scholar, Europe PMC, Medline, and Scopus databases. Continuous variables were combined using mean difference (MD), while binary variables were analysed using risk ratio (RR) with 95% confidence intervals through random-effects models.

**Results:**

Our analysis included nine studies. The results showed that EUC was associated with a significantly lower incidence of stent-related symptoms [RR 0.32 (95% CI 0.19 - 0.54), p < 0.0001, I² = 24%] compared to the DJ-stent. There were no significant differences between EUC and DJ-stent in terms of postoperative fever (p = 0.92), urine leakage (p = 0.21), perinephric collection (p = 0.85), haemoglobin drop (p = 0.06), transfusion rate (p = 0.27), VAS score (p = 0.67), analgesic requirements (p = 0.59), stone-free rate (p = 0.14), duration of surgery (p = 0.10), and duration of hospitalization (p = 0.50).

**Conclusion:**

The EUC demonstrated fewer stent-related symptoms than the DJ-stent in tubeless PCNL, while both methods showed comparable safety and efficacy. The choice between EUC and DJ-stent should consider patient preferences and surgeon expertise. Further randomized controlled trials (RCTs) with larger sample sizes are needed to affirm these results.

## INTRODUCTION

Urolithiasis is a common urological condition, with over 115 million cases globally and a prevalence ranging from 1% to 13% across different regions.(1) Percutaneous nephrolithotomy (PCNL) is a cutting-edge procedure for stone removal via percutaneous access and has become the preferred treatment for renal stones larger than two cm or those unresponsive to extracorporeal shock wave lithotripsy (2-4).

Traditionally, PCNL involves using a nephrostomy tube to maintain urinary drainage, control bleeding, and provide access for additional procedures if needed (5, 6). In 1997, Bellman introduced a modified technique using a double-J stent (DJ-stent), referred to as tubeless PCNL.(6, 7) This technique has been further modified by leaving an externalized ureteral catheter overnight. A meta-analysis of 14 randomized controlled trials (RCTs) demonstrated that tubeless PCNL reduces hospital stay duration, shortens recovery time, lowers postoperative pain scores, and decreases urine leakage compared to standard PCNL (8).

Despite these advantages, DJ-stents are associated with several adverse events and need to be removed after a few weeks, causing additional distress and costs for patients (9). Conversely, external ureteral catheters (EUCs) often result in fewer postoperative complaints, are easier to remove without additional distress, and do not incur extra costs (10, 11).

The literature comparing EUC and DJ-stent in tubeless PCNL shows conflicting results. An RCT by Telha KA et al. found lower postoperative complications with DJ-stent compared to EUC in tubeless PCNL.(10) In contrast, an RCT by Habib B et al. reported fewer stent-related symptoms in patients using EUC compared to those using DJ-stents.(11) Given these inconsistencies, a meta-analysis is necessary to clarify the comparative efficacy of EUC and DJ-stent as drainage methods in tubeless PCNL. This study aims to consolidate the latest evidence on this comparison.

## MATERIALS AND METHODS

### Eligibility Criteria

The study protocol was registered in the PROSPERO database, number CRD42023415836. This review follows the PRISMA statement and Cochrane Handbook guidelines (12,13). Included studies met these criteria: (1) adult patients with upper urinary tract (kidney and ureter) calculi treated with tubeless percutaneous nephrolithotomy (PCNL) (Population); (2) comparison between external ureteral drainage (EUC) and double-J stent (DJ-stent) in tubeless PCNL (Intervention and Control); (3) data on stent-related symptoms, postoperative fever, urine leakage, perinephric collection (urinoma, perinephric abscess, perirenal hematoma), haemoglobin drop, transfusion rate, postoperative visual analog scale (VAS) scores, analgesic requirements, stone-free rate, surgery duration, and hospitalization duration (Outcome); and (4) observational studies (cohort/case-control) or randomized clinical trials (RCTs) (Study Design). Excluded studies included: (1) on pediatric populations; (2) using standard (non-tubeless) PCNL; (3) presented as case reports, case series, or review articles; and (4) not available in full-text.

### Literature Search and Study Selection

Two independent authors searched English literature in Europe PMC, Scopus, Medline, and ClinicalTrials.gov until July 15, 2023, using combined key words: "(ureteral catheter OR ureteric catheter OR external ureteral catheter OR EUC OR ureteral stent) AND (double J stent OR DJ-stent OR double pigtail stent) AND (percutaneous nephrolithotomy OR tubeless percutaneous nephrolithotomy OR PCNL)". After removing duplicates, titles and abstracts were screened, and full-text evaluations were performed on articles passing the initial screening to ensure they met inclusion criteria. Discrepancies were resolved by a third author.

### Data Extraction and Quality Assessment

Data descriptions including author names, publication year, study design, sample size, baseline characteristics (mean age, sex distribution, stone location, stone size/burden, affected side), and outcomes were collected. Two independent authors tabulated the data into Microsoft Excel 2019. Risk of bias was evaluated using the Cochrane Collaboration's Risk of Bias version 2 (RoB v2) instrument for RCTs, evaluating randomization, deviations from intended interventions, outcome measurement, and missing outcome data. Evaluations were categorized as "low risk," "high risk," or "some concerns" (14). For cohort/case-control studies, the Newcastle-Ottawa Scale (NOS) from the Ottawa Hospital Research Institute (OHRI) was used, assessing participant selection, comparability, and outcome ascertainment, with scores ≥7 indicating "good" quality (15).

### Statistical Analysis

Common mean difference (MD) of 95% confidence intervals was used to pool continuous outcomes using the Inverse-Variance formula. For haemoglobin drop, we used standardized mean difference (SMD) due to data expression variations. Dichotomous outcomes were pooled into risk ratio (RR) with 95% CI by the Mantel-Haenszel formula. Random-effect models were used due to expected heterogeneity. Heterogeneity was assessed with the I-squared (I²) statistic, with I² > 50% indicating significant heterogeneity. Data expressed as medians and interquartile ranges (IQR) or as medians with minimum and maximum values were converted to means and standard deviations (SD) using formulas from Wan X et al. and Luo D et al (16,17). Publication bias analysis was performed when more than 10 studies were available for an outcome. We used Review Manager 5.4 from the Cochrane Collaboration as the main software for statistical analysis of this study.

## RESULTS

### Study Selection and Characteristics

**Figure 1:**
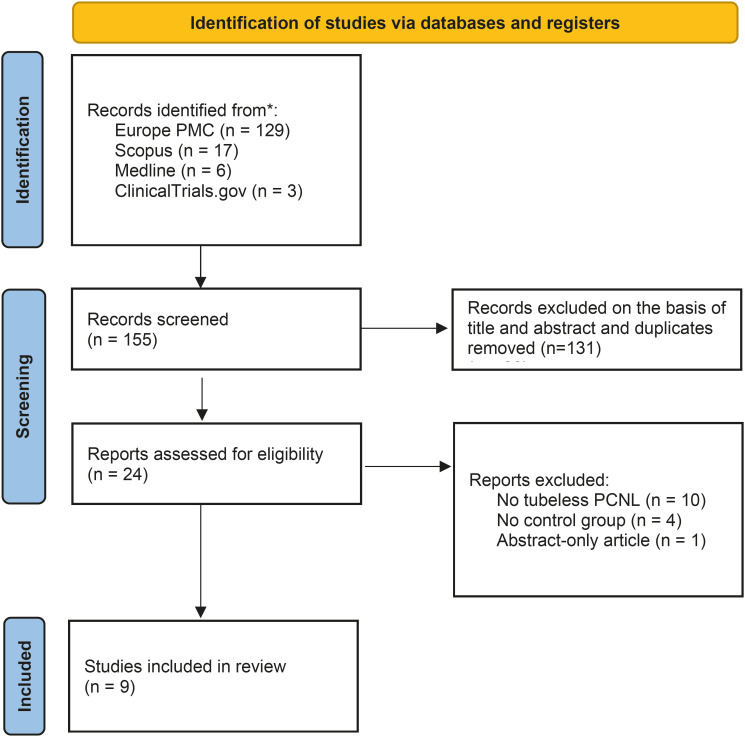
PRISMA diagram of the detailed process of selection of studies for inclusion in the systematic review and meta-analysis.

A search across four international databases identified 155 studies. After screening and eliminating duplicate studies, 131 studies were successfully excluded, leaving only 24 studies for further assessment in full-text. Of these, 15 studies were eventually ruled out for the following reasons: 10 used standard (non-tubeless) PCNL as the comparison, 4 lacked a control group, and 1 was only an abstract. Hence, there were only 9 studies included in the final analysis (Figure-1) (10, 11, 18-24). Of these, 6 were prospective RCTs and 3 were retrospective observational studies. Sample sizes ranged from 23 to 227 in the EUC group and 23 to 189 in the DJ-stent group. Stone locations included the renal pelvis, renal calyx, upper ureter, and staghorn calculi, with mean stone sizes from 1.6 to 9.1 cm. Both EUC and DJ-stent insertions were performed immediately after the procedure. Data on catheter or stent removal timing were not described. Baseline characteristics of the included studies are outlined in Table-1.

**Table 1:** Characteristic of Included Study.

Authors	Study Design	Tubeless PCNL with EUC						Tubeless PCNL with DJ-stent					
		Sample size	Age (mean ± SD)	Male (%)	Stone location*	Stone measurement (mean ± SD)	Affected side	Sample size	Age (mean ± SD)	Male (%)	Stone location*	Stone measurement (mean ± SD)	Affected side
Randomized studies													
Gonen M et al.[18] 2009	Prospective RCT	23	44.5 ± 14.7	52.1%	P: 21.7% C: 34.7% P + C: 21.7% UU: 4.3% S: 17.3%	Burden: 909 ± 882 mm2	R: 52.1% L: 47.9%	23	51.7 ± 13.9	47.9%	P: 30.4% C: 30.4% P + C: 8.7% UU: 17.4% S: 13%	Burden: 765.2 ± 610.7 mm2	R: 47.9% L: 52.1%
Habib B et al.[11] 2022	Prospective RCT	40	41.5 ± 17.5	42.5%	NR	Size: 1.9 ± 0.4 cm	R: 45% L: 55%	40	39.7 ± 11.7	52.5%	NR	Size: 2.4 ± 0.6 cm	R: 42.5% L: 57.5%
Jiang H et al.[19] 2017	Prospective RCT	30	45.9 ± 11.4	46.7%	NR	Burden: 166 ± 78.7 mm2	NR	30	49.4 ± 15.5	30%	NR	Burden: 169 ± 94.1 mm2	NR
Mercado A et al.[20] 2013	Prospective RCT	35	48.9 ± 9	48.5%	NR	Burden: 4.9 ± 1.7 cm2	NR	33	52.6 ± 11.9	51.5%	NR	Burden: 5.8 ± 2.8 cm2	NR
Telha KA et al.[10] 2010	Prospective RCT	76	29 ± 8.9	73.6%	NR	Size: 4.3 ± 0.6 cm	R: 47.3% L: 52.7%	72	31 ± 9.2	75%	NR	Size: 4.9 ± 0.7 cm	R: 59.7% L: 40.3%
Zhou Y et al.[21] 2016	Prospective RCT	56	51.3 ± 12.6	55.4%	P: 42.9% C: 28.6% UU: 10.7% M: 17.8%	Size: 21.8 ± 9.19 mm2	NR	53	48.6 ± 14.8	54.7%	P: 49.1% C: 32% UU: 7.5% M: 11.4%	Size: 22.5 ± 8.58 mm2	NR
Observational studies													
Chung HS et al.[22] 2016	Retrospective cohort	61	56.6 ± 13.2	62.3%	P + C: 60.6% UU: 4.9% S: 23% M: 9.8%	Size: 4.2 ± 1.1 cm	R: 37.7% L: 62.3%	108	54.5 ± 12.6	58.3%	P + C: 54.6% UU: 3.7% S: 36.1% M: 6.4%	Size: 4.2 ± 1.4 cm	R: 41.6% L: 58.4%
Gonulalan U et al.[23] 2013	Retrospective cohort	148	46.8 ± 14.2	59.4%	P: 19.6% C: 43.9% P + C: 30.4% UU: 4.1% S: 2%	Burden: 33.8 ± 1.7 mm2	NR	120	46.8 ± 14.2	59.4%	P: 19.6% C: 43.9% P + C: 30.4% UU: 4.1% S: 2%	Burden: 35.6 ± 1.5 mm2	NR
Raharja PAR et al.[24] 2019	Retrospective cohort	227	50.7 ± 11.2	58.1%	P: 31.3% C: 27.8% P + C: 37.9% UU: 3.1%	Burden: 33.4 ± 17.2 mm2	R: 49.3% L: 50.7%	189	50.3 ± 11.6	63.5%	P: 32.3% C: 28% P + C: 38.1% UU: 1.6%	Burden: 36.1 ± 22.1 mm2	R: 46% L: 54%

DJ-stent = double J stent; EUC = external ureteral catheter; NR = not reported; PCNL = percutaneous nephrolithotomy; RCT = randomized clinical trial; R/L = right/left; SD = standard deviation

* Stone location: P = pelvis; C = caliceal; P + C = pelvis and caliceal; UU = upper ureter; S = staghorn; M = multiple locations

### Quality of Study Assessment

The assessment for the bias risk using the RoB v2 instrument found that two of the six RCTs had a "low risk" of bias across all five domains (19, 21). The other four RCTs were rated as having "some concern" due to insufficient information on allocation concealment post-randomization (10, 11, 18, 20), despite appropriate randomization methods and balanced baseline characteristics. The NOS tool assessed all cohort studies as "good quality" with scores of 8. The risk of bias assessments is summarized in Table-2.

**Table 2:** Risk of Bias assessment of the included studies using RoB v2 tool.

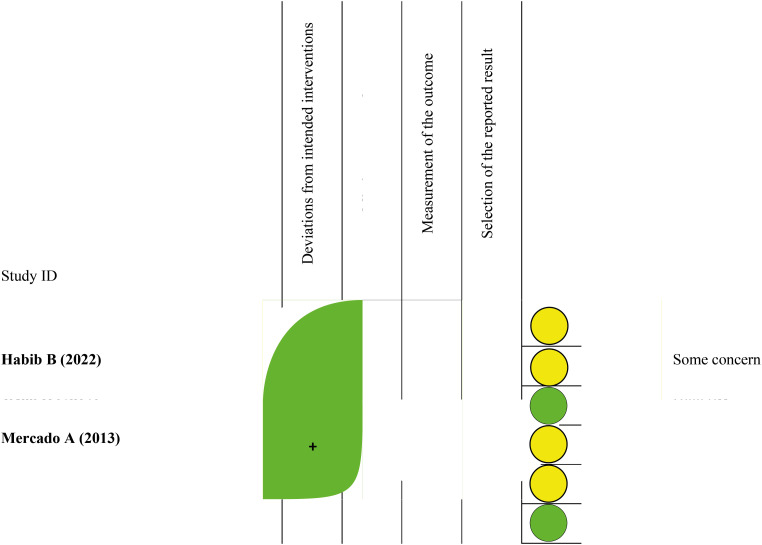

### OUTCOMES OF INTEREST

#### Stent-Related Symptoms

**Figure 2:**
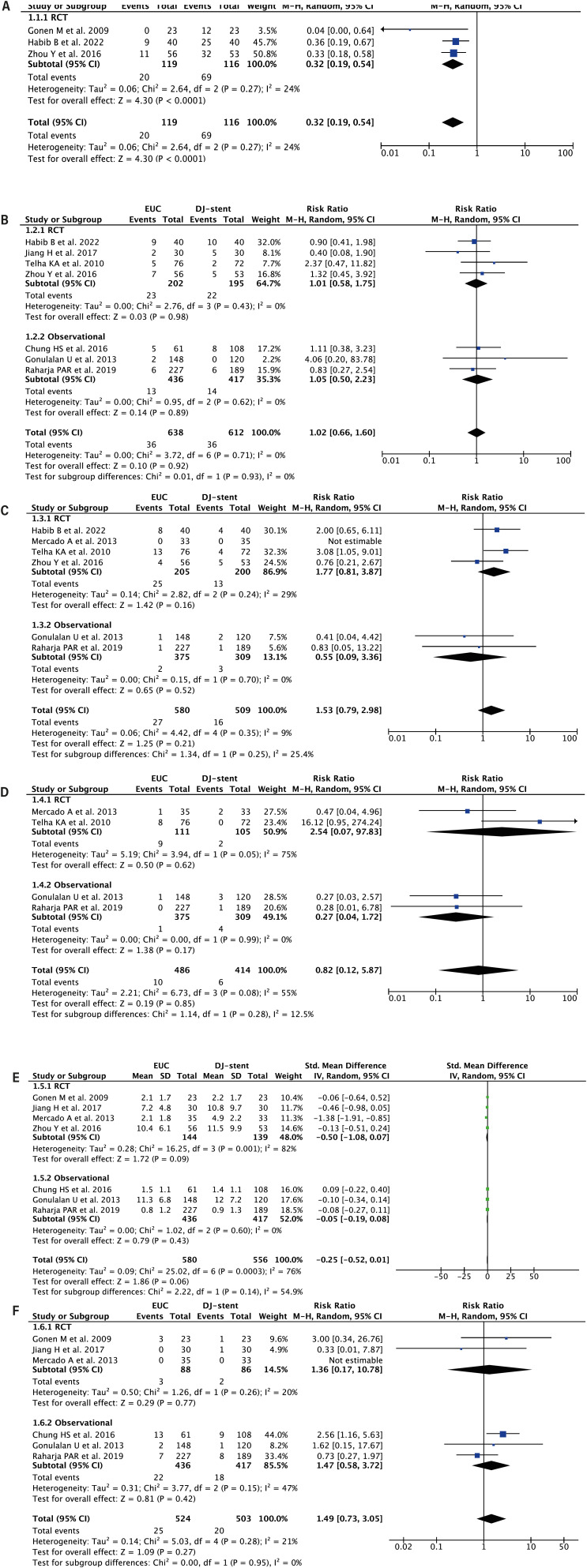
Forest plot that demonstrates the comparison between EUC vs DJ-stent in tubeless PCNL in terms of: Stent-related symptoms (A), Postoperative fever (B), Urine leak (C), Perinephric collection (D), Haemoglobin drop (E), Transfusion Rate (F).

Pooled analysis from 3 RCTs (n = 235) showed that EUC was associated with a lower risk of stent-related symptoms compared to DJ-stent [RR 0.32 (95% CI 0.19 - 0.54), p < 0.0001, I² = 24%] (Figure-2A).

#### Post-Operative Fever

Pooled analysis of 7 studies (n = 1,250) found no significant difference in post-operative fever rates between EUC and DJ-stent groups [RR 1.02 (95% CI 0.66 - 1.60), p = 0.92, I² = 0%] (Figure-2B). Subgroup analysis by study design confirmed non-significant results for both RCTs (p = 0.98) and observational studies (p = 0.89).

#### Urine Leakage

Pooled analysis from 6 studies (n = 1,089) revealed no significant difference in urine leakage between EUC and DJ-stent methods [RR 1.53 (95% CI 0.79 - 2.98), p = 0.21, I² = 9%] (Figure-2C). Subgroup analysis by study design showed non-significant results for both RCTs (p = 0.16) and observational studies (p = 0.52).

#### Perinephric Collection

Pooled analysis from 4 studies (n = 900) found no significant difference in perinephric collection between EUC and DJ-stent methods [RR 0.82 (95% CI 0.12 - 5.87), p = 0.85, I² = 55%] (Figure-2D). Subgroup analysis showed non-significant results for both RCTs (p = 0.62) and observational studies (p = 0.17).

#### Haemoglobin Drop

Pooled analysis from 7 studies (n = 853) showed no significant difference in haemoglobin drop between EUC and DJ-stent methods [SMD −0.25 (95% CI −0.52, 0.01), p = 0.06, I² = 76%] (Figure-2E). Subgroup analysis showed non-significant results for both RCTs (p = 0.09) and observational studies (p = 0.43).

#### Transfusion Rate

Pooled analysis from 6 studies (n = 1,027) found no significant difference in transfusion rates between EUC and DJ-stent methods [RR 1.49 (95% CI 0.73 - 3.05), p = 0.27, I² = 21%] (Figure-2F). Subgroup analysis showed non-significant results for both RCTs (p = 0.77) and observational studies (p = 0.42).

#### Visual Analog Scale (VAS)

**Figure 3:**
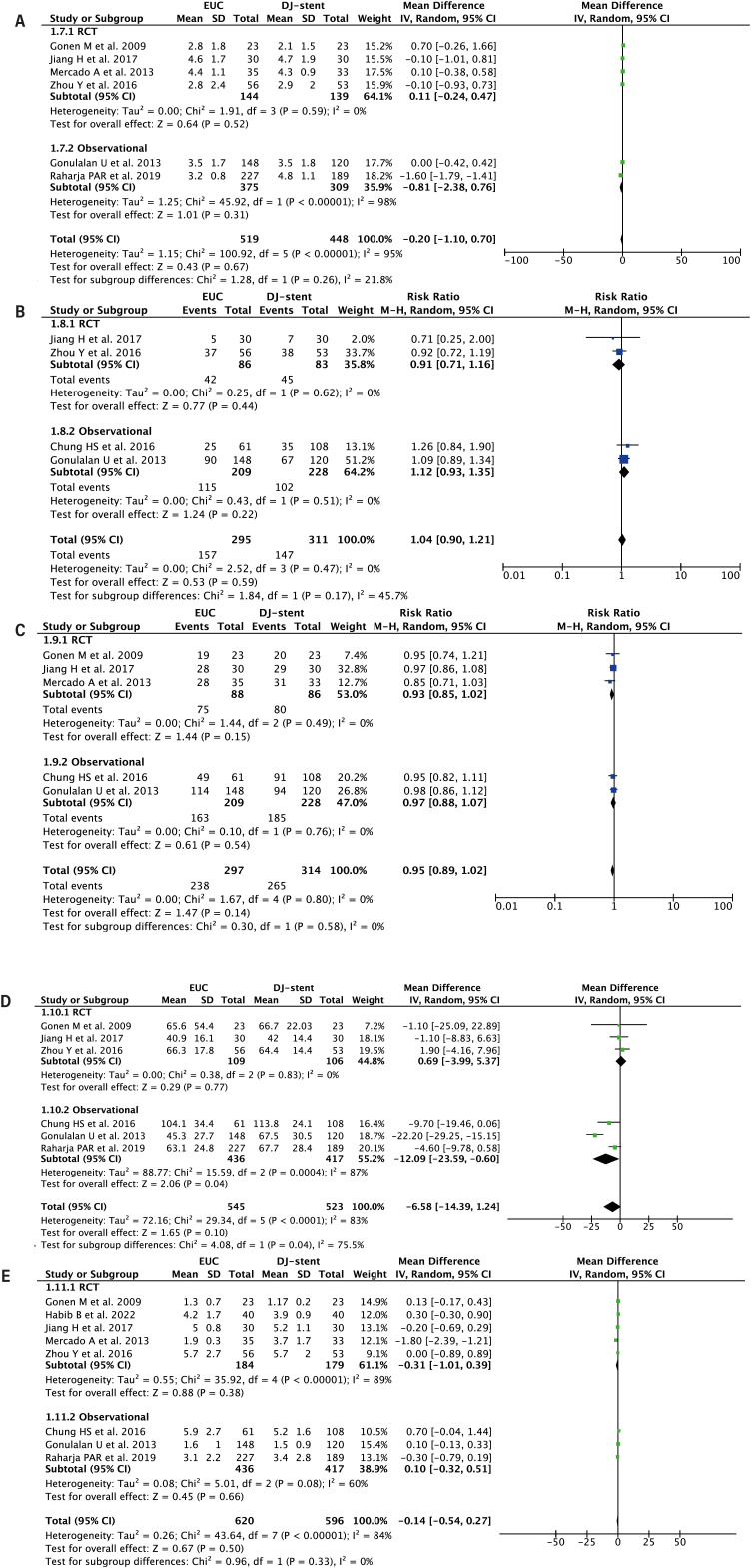
Forest plot that demonstrates the comparison between EUC vs DJ-stent in tubeless PCNL in terms of: VAS score (A), Analgesic requirement (B), Stone free rate (C), Duration of surgery (D), Duration of hospitalization (E).

Pooled analysis from 5 studies (n = 611) showed no significant difference in post-operative VAS scores between EUC and DJ-stent methods [MD −0.20 (95% CI −1.10, 0.70), p = 0.67, I² = 95%] (Figure-3A). Subgroup analysis showed non-significant results for both RCTs (p = 0.52) and observational studies (p = 0.31).

#### Analgesic Requirements

Pooled analysis from 5 studies (n = 611) revealed no significant difference in analgesic requirements between EUC and DJ-stent methods [RR 1.04 (95% CI 0.90 - 1.21), p = 0.59, I² = 0%] (Figure-3B). Subgroup analysis showed non-significant results for both RCTs (p = 0.44) and observational studies (p = 0.22).

#### Stone-Free Rate

Pooled analysis from 5 studies (n = 611) demonstrated no significant difference in stone-free rates between EUC and DJ-stent methods [RR 0.95 (95% CI 0.89 - 1.02), p = 0.14, I² = 0%] (Figure-3C). Subgroup analysis showed non-significant results for both RCTs (p = 0.15) and observational studies (p = 0.54).

#### Duration of Surgery

Pooled analysis from 6 studies (n = 1,068) showed no significant difference in surgery duration between EUC and DJ-stent methods [MD −6.58 min (95% CI −14.39, 1.24), p = 0.10, I² = 83%] (Figure-3D). Subgroup analysis showed significant results for observational studies but with high heterogeneity [MD −12.09 min (95% CI −23.59, −0.60), p = 0.04, I² = 87%], while results for RCTs remained non-significant with low heterogeneity (p = 0.77, I² = 0%).

#### Duration of Hospitalization

Pooled analysis from 8 studies (n = 1,216) showed no significant difference in hospitalization duration between EUC and DJ-stent methods [MD −0.14 days (95% CI −0.54, 0.27), p = 0.50, I² = 84%] (Figure-3E). Subgroup analysis showed non-significant results for both RCTs (p = 0.38) and observational studies (p = 0.66).

#### Publication Bias

Bias analysis was not conducted because there were less than 10 studies available for each outcome. It makes both funnel plots and statistical tests to detect the publication bias to be less reliable.(25,26)

## DISCUSSION

Our study demonstrates that using an external ureteral catheter (EUC) for drainage in tubeless percutaneous nephrolithotomy (PCNL) is associated with fewer stent-related symptoms compared to a double-J stent (DJ-stent) (25). However, no significant differences were found between EUC and DJ-stent regarding postoperative complications, visual analog scale (VAS) scores, analgesic requirements, stone-free rates, surgery duration, or hospitalization duration (25).

These findings are consistent with those of the previous meta-analysis by Chen Y et al., which also found that EUC had fewer stent-related symptoms than DJ-stent (25). Other outcomes, such as surgery duration and postoperative complications, showed no significant differences between the two methods. However, there are several important distinctions between our study and the meta-analysis conducted by Chen Y et al. (25).

First, our study included nine studies (six RCTs and three cohort studies), whereas Chen Y et al. included only seven studies (five RCTs and two non-RCTs) (25). By including more studies, our analysis provides a stronger evidence base and potentially more reliable conclusions.

Second, Chen Y et al. combined data from RCTs and non-RCTs in their analysis, which is not recommended by the Cochrane Handbook due to the potential biases inherent in observational studies (13, 25). Observational studies are susceptible to selection bias and information bias, which can impact the validity of the results.(13,26) Selection bias can lead to differences in baseline characteristics, and information bias can reduce data validity (26). RCTs minimize these biases through randomization and allocation concealment (27, 28). Our study adhered to Cochrane guidelines by separating the results of RCTs from those of observational studies, thereby ensuring more reliable findings (13).

Third, Chen Y et al. grouped postoperative complications into major and minor categories, potentially obscuring specific differences (25). Our study categorized complications into distinct types, such as postoperative fever, urine leakage, perinephric collection, haemoglobin drop, and transfusion rate, providing a clearer and more detailed comparison. Consequently, our study assessed 11 outcomes compared to Chen Y et al.'s nine (25).

The choice between EUC and DJ-stent for PCNL should consider their respective advantages and disadvantages. Our analysis highlights that EUC is associated with fewer stent-related symptoms and may be more cost-effective and practical, especially in resource-limited settings. EUC is easier to remove and more economical, which can be particularly beneficial for patients in developing countries or those with limited resources (29, 30). Despite fewer stent-related symptoms, the external ureteral catheter (EUC) does come with its own set of complications, notably spontaneous removal and displacement. These complications can lead to unexpected patient discomfort and the need for additional medical interventions. Given these potential issues, it is crucial for surgeons to carefully access clinical and physical condition of the patient.

DJ-stents offer specific benefits, such as maintaining ureteral patency, preventing obstruction, and facilitating the clearance of stone fragments. Despite their higher initial costs and potential discomfort, DJ-stents may be preferred in cases with complex stone burdens or challenging ureteral anatomy.(30) Ultimately, the decision should be based on individual patient characteristics, surgeon preference, and resource availability.

Endoscopic combined intrarenal surgery (ECIRS) is an emerging technique that combines retrograde and antegrade approaches for stone management (31). The increasing adoption of ECIRS has implications for the use of DJ-stents post-surgery, due to their ability to maintain ureteral patency and prevent obstruction, which is particularly important when the ureter is manipulated extensively leading to local edema (32). Although our study focused on tubeless PCNL and did not evaluate ECIRS, it is crucial to acknowledge that the findings from our study may not be directly applicable to ECIRS. Future research should specifically address the outcomes and stent-related complications in the context of ECIRS to guide clinical practice accurately.

Our study has some limitations. The number of included studies, particularly the RCTs, are relatively small, with fewer than 100 participants in many cases. This may limit the generalizability of the results. Additionally, significant heterogeneity was observed in some outcomes, such as haemoglobin drop, VAS score, surgery duration, and hospitalization duration. This heterogeneity is likely influenced by variations in surgeon experience, surgical techniques, and outcome definitions. Differences in stone size and location across studies also contribute to this heterogeneity. Further well-designed larger RCTs are needed to affirm our findings. Moreover, data on long-term complications and readmission rates are lacking and should be addressed in future research.

## CONCLUSION

Our study suggests that EUC results in fewer stent-related symptoms than DJ-stent in tubeless PCNL and is comparable in terms of postoperative complications, pain, surgery duration, and hospitalization. The choice between EUC and DJ-stent should be based on patient preference and surgeon judgment, considering individual risks and benefits. Future RCTs are recommended to validate our findings in this study.
